# Genetic Structure Is Associated with Phenotypic Divergence in Floral Traits and Reproductive Investment in a High-Altitude Orchid from the Iron Quadrangle, Southeastern Brazil

**DOI:** 10.1371/journal.pone.0120645

**Published:** 2015-03-10

**Authors:** Bruno Leles, Anderson V. Chaves, Philip Russo, João A. N. Batista, Maria Bernadete Lovato

**Affiliations:** 1 Departamento de Biologia Geral, Instituto de Ciências Biológicas, Universidade Federal de Minas Gerais, Belo Horizonte, Minas Gerais, Brasil; 2 Departamento de Botânica, Instituto de Ciências Biológicas, Universidade Federal de Minas Gerais, Belo Horizonte, Minas Gerais, Brasil; Università Politecnica delle Marche, ITALY

## Abstract

Knowledge of the role of Neotropical montane landscapes in shaping genetic connectivity and local adaptation is essential for understanding the evolutionary processes that have shaped the extraordinary species diversity in these regions. In the present study, we examined the landscape genetics, estimated genetic diversity, and explored genetic relationships with morphological variability and reproductive strategies in seven natural populations of *Cattleya liliputana* (Orchidaceae). Nuclear microsatellite markers were used for genetic analyses. Spatial Bayesian clustering and population-based analyses revealed significant genetic structuring and high genetic diversity (He = 0.733 ± 0.03). Strong differentiation was found between populations over short spatial scales (FST = 0.138, p < 0.001), reflecting the landscape discontinuity and isolation. Monmonier´s maximum difference algorithm, Bayesian analysis on STRUCTURE and principal component analysis identified one major genetic discontinuity between populations. Divergent genetic groups showed phenotypic divergence in flower traits and reproductive strategies. Increased sexual reproductive effort was associated with rock outcrop type and may be a response to adverse conditions for growth and vegetative reproduction. Here we discuss the effect of restricted gene flow, local adaptation and phenotypic plasticity as drivers of population differentiation in Neotropical montane rock outcrops.

## Introduction

Tropical mountains play an important role in promoting regional and global biodiversity [1]. Montane ecosystems contain some of the world´s richest plant communities [2–4], but our knowledge about the evolutionary patterns of plant diversity in these regions remains poor [5–8]. Nonetheless, the risk of habitat loss under climate change would lead to high extinction rates for plants and animals in these regions [9–12].

Tropical mountains are important models to test species radiation and the effects of landscape properties on genetic structure of natural populations [13–21]. Tropical montane landscapes combine discontinuous distribution with edapho-climatic variations resulting from altitudinal gradients. Because of their disconnected geographic nature, rock outcrops are frequently compared with oceanic islands, since most of them display a marked ecological isolation from the surrounding area. Indeed, geographic isolation on islands has proven to be a useful model system for the study of species’ radiation [22–25]. The basic concept and tools developed to study island biogeography, along with new methodological developments to include spatial and temporal data regarding population genetics and genomics, can be applied to evolutionary studies in this context. This approach would provide insights on mechanisms and patterns of species diversification [26–30]. Moreover, understanding the pattern of genetic connectivity, gene flow and local adaptations has clear implications for the proper management of genetic diversity in threatened populations [30,31].

**Fig 1:**
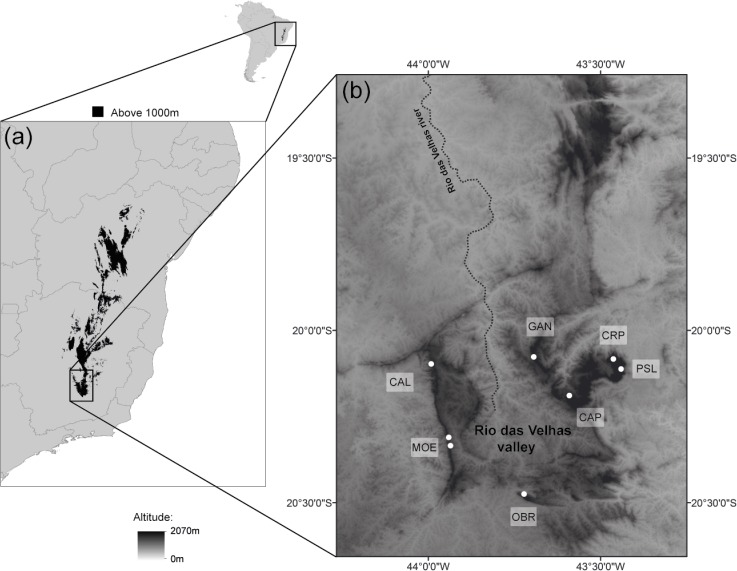
Sampled populations of *Cattleya liliputana* occurring in the Iron Quadrangle. (a) Espinhaço Range Region above 1,000 m a.s.l. in eastern tropical South America. (b) Altitude map of the Iron Quadrangle showing the *Cattleya liliputana* populations analysed in the study.

The Espinhaço Range Region (ERR) is an orographic formation that runs north-south over 1,000 km in the eastern tropical region of South America where there are high numbers of rock outcrop formations. Espinhaço Mountains are at the edge of important biomes, such as the Atlantic Rain Forest, Cerrado, and Caatinga. The Iron Quadrangle (IQ) makes up the southern end of the ERR and covers an area of approximately 7,200 km^2^ in Minas Gerais state, southeastern Brazil (Fig. 1). The IQ is known for its high geological heterogeneity and represents one of the most important and well-studied geological sites in South America [32,33], being responsible for around 50% of Brazilian iron ore production [34]. Quartzite and iron rock outcrops are interspersed throughout the tops of the IQ Mountains. Outcrops typically harbour rupicolous vegetation embedded within a landscape composed of contrasting plant communities. Studies of plant communities at iron outcrops have revealed that most of the species are highly structured by the presence of outcrops and the availability of specific microhabitat conditions, resulting in high indices of α and β diversity [35]. The IQ is recognized as a region with high floristic diversity and endemism in the South American highlands, containing over 30% of endemic species, most of which are associated with rock outcrop environments [8,36].

The isolation of rock outcrop species is expected to constrain gene flow, enhancing the effects of genetic drift, selection, and population divergence [37,38]. Divergent natural selection based on contrasting environments can promote phenotypic and genetic differentiation among populations [39,40], resulting in local adaptive radiation. The edaphic structure (occurrence of iron and quartzite rock outcrops) and climatic heterogeneity of the IQ make it an interesting model to study the role of contrasting landscape and environmental factors on intraspecific genetic and ecological divergence in Neotropical plants.

In the present study, we examined the landscape genetics, morphological variation, and reproductive investment of *Cattleya liliputana* (Pabst) van den Berg, a rupicolous orchid restricted to high-altitude rock outcrops in the IQ. The objectives were: (i) to test how landscape factors affect genetic structure and the degree of isolation among disjointed populations; (ii) to test whether genetic structure is associated with divergence in floral and vegetative morphological traits and reproductive investment; and, (iii) to test whether genetic, morphological and reproductive differentiation is associated with climatic and edaphic factors (iron or quartzite outcrops). The results showed that genetic divergence is associated with landscape and edaphic factors. The genetically differentiated populations exhibited morphological and reproductive divergence. These genetic and ecological divergences are likely to influence the long-term success of conservation projects and may aid our understanding of the major evolutionary processes in *Cattleya* radiation in Neotropical montane habitats.

## Materials and Methods

### Ethics Statement

The collection of the samples in the private conservation units RPPN Santuário do Caraça and RPPN Capanema were realized with authorization of local administrators (Pe. Wilson Belloni, director of Santuário do Caraça and the Department of Environmental and Sustainable Development—Vale S.A.). The collection of samples out of conservation units were realized with the legal authorization of the Instituto Brasileiro do Meio Ambiente e dos Recursos Naturais Renováveis IBAMA (Sisbio #23771).

### Study species and population sampling

*Cattleya liliputana* (Pabst) van den Berg [41,42] is a rupiculous orchid that is restricted in its distribution to the Iron Quadrangle. The species can be identified in the field by small reddish leafs (9 ± 2.5 mm) and a small round lip (around 10 × 10 mm) with a yellow mid lobe (S1 and S2 Figs.) [43,44]. Individuals produce usually 1–5 inflorescences bearing 1–3 flowers from July to October. Flowers usually last for several days, about 20% of flowers are pollinated, and usually less than 5% of individuals produce fruits (B. Leles, unpublished data). Fruits open between November and December, and seeds are dispersed by the wind.

Samples of leaves for DNA extraction, and pseudobulbs bearing inflorescences for morphological measurements, were collected from seven populations in the major mountain ranges of the Iron Quadrangle (IQ) (Fig. 1). The populations of *C*. *liliputana* studied are found in iron and quartzite rock outcrops from 1483 to 2055 m a.s.l. Five populations (CAL, MOE, OBR, CAP, and GAN) are found around the Rio das Velhas river valley having a U-shaped distribution. This region is characterised by large iron ore deposits and intensive mining activities. Populations grow predominantly on iron outcrops and are rarely found on small quartzite outcrops embedded in iron deposits. Two other populations (CRP and PSL) are located in a peripheral mountain named Serra do Caraça (Fig. 1, Table 1). This region is characterized by large and massive quartzite outcrops reaching the highest altitudes of the ERR mountains. A minimum distance of five meters between individuals was established during population sampling.

**Table 1:** Population code, location, type of substrate, sample size and voucher information of studied populations of *Cattleya liliputana* (N = 160 individuals) from the Iron Quadrangle, Minas Gerais state, southeastern Brazil.

Code	Mountain Range	Municipality	Coordinates	Rock outcrop	Altitude	*N*_gentics_	*N*_morph_.	Voucher
CAL	Serra da Calçada	Brumadinho	20°05'S/43°56'W	Iron	1483	24	13	BHCB64807
MOE	Serra da Moeda	Moeda	20°20'S/43°56'W	Iron	1530	24	23	B. Leles 002
OBR	Serra de Ouro Branco	Ouro Branco	20°29'S/43°52'W	Quartzite	1513	24	7	B. Leles 082
CAP	Serra de Capanema	Ouro Preto	20°12'S/43°54'W	Iron	1807	24	17	BHCB112460
GAN	Serra da Gandarela	Rio Acima	20°05'S/43°41'W	Iron	1611	28	19	B. Leles 001
CRP	Serra do Caraça	Catas Altas	20°04'S/43°28'W	Quartzite	1637	20	11	B. Leles 057
PSL	Serra do Caraça	Catas Altas	20°06'S/43°27'W	Quartzite	2055	16	21	BHCB47268

Vouchers are deposited in the herbarium BHCB

### DNA extraction, PCR, and genotyping

Total DNA was extracted following the protocol described by Doyle and Doyle [45]. Seven microsatellite markers isolated for *Cattleya coccinea* [46,47] were used to analyse genetic diversity and landscape genetics of *Cattleya liliputana* (S1 Table).

The amplification reactions were performed in a final volume of 25 μL containing 1 unit of Taq polymerase (Phoneutria), 1 × IB Phoneutria buffer with 1.5 mM MgCl_2_, 0.2 mM dNTP, 0.04 to 0.16 μM primers, 0.16 μM HEX or FAN-labelled M13 primers, and 10 ng of genomic DNA. Reverse amplification primers containing an M13 sequence were used as a fluorescent label, according to the method used by Schuelke [48], (S1 Table). PCR cycles were as follows: premelting at 94°C for 5 min, 10 cycles of denaturation at 95°C for 30 sec, an annealing phase at 56–60°C (depending on the primer) for 1 min, an extension phase at 72°C for 1 min, followed by 25 cycles with denaturing at 89°C for 30 sec, an annealing phase at 53°C for 1 min, an extension phase at 72°C for 1 min a final extension at 72°C for 45 min. Genotyping was performed on a MegaBACE 1000 automated sequencer, using 0.1% Tween 20 and ROX-500 size standards (GE Healthcare). Alleles were identified using MegaBACE Fragment Profiler version 1.2 software (GE Healthcare). The presence of null alleles and scoring errors at each locus was tested using MICRO-CHECKER version 2.2.3 [49] For microsatellite primers and amplification conditions see S1 Table.

### Genetic diversity and landscape genetics

Observed (H_o_) and expected (H_e_) heterozygosities, the number of alleles, and deviations from Hardy–Weinberg equilibrium were estimated for each locus and each population using the software Arlequin, version 3.1 [50]. Allelic richness (A_R_) with rarefaction and *F*_IS_ estimates were performed in FSTAT 2.9.3 [51].

Population structure and landscape genetics were analysed using several methods. Arlequin 3.1 was used to perform Analysis of Molecular Variance (AMOVA). Three AMOVAs were performed. One considered only two hierarchical levels and analysed the partition of total genetic diversity between and within populations. Two AMOVAs tested for barriers to gene flow. One AMOVA tested differentiation between two groups according to software STRUCTURE 2.3.4 [52] and principal component analysis (PCA): the Serra do Caraça populations (CRP and PSL) and core IQ populations (CAL, MOE, OBR, CAP, and GAN). AMOVA was also used to test for groupings according to neighbour-joining analysis, considering three groups: Serra do Caraça populations and the division of core populations into a western group (CAL, MOE, and OBR) and an eastern group (CAP and GAN).

Bayesian analysis of population structure was performed using STRUCTURE [52], and models consisted of ten independent runs for each K, set from K = 1 to K = 10, consisting of 1,000,000 Markov chain Monte Carlo (MCMC) iterations, with an initial burn-in of 50,000, allowing admixture and assuming correlated allele frequencies. To determine the optimum number of clusters, we calculated the average likelihood of each K, ‘log of probability’ (LnP(D)), through all runs as suggested by Pritchard *et al*. [52] and the ΔK statistic according to Evanno *et al*. [53]. The occurrence of barriers between populations was tested using Monmonier´s maximum difference algorithm implemented by Alleles in Space [54].

The number of populations, and the occurrence of genetic discontinuities among them, were also inferred by a spatial explicit Bayesian model based on simulations of microsatellite data and geographic information carried out in Geneland [55,56]. MCMC simulations consisted of 20,000,000 interactions with a thinning of 2,000, with correlated allele frequencies and corrections for null alleles. At least ten independent runs were performed and the best model was selected based on posterior probability with a burn-in of 1,000. To characterise the spatial distribution of genetic populations defined by Bayesian simulations, maps of population membership probability for each designed cluster were generated. Population structure was further analysed by performing PCA based on the allelic frequencies for each population. PCA was performed using the *adegenet* package [57] for R 2.15.1 [58].

### Morphological and reproductive investment analyses

Flowering pseudobulbs were collected in the field and preserved in 70% ethanol. Flowers were dissected under a stereo microscope, mounted on plastic cover slips with a reference scale, and scanned at 600 dpi. Dissected pseudobulbs were measured with a digital calliper. Digitalized images of flower parts were measured with software ImageTool 3.0. Thirty-two continuous characters, 24 floral and eight vegetative (S2 Fig.), were measured. Measurements were Ln-transformed, and correlation analysis was used to identify autocorrelation between variables. Variables with autocorrelation higher than 85% were excluded from analysis. A discriminant analysis was conducted for 26 remaining characters performed with population as the categorical variable. Cluster analysis of the populations was performed using the Mahalanobis generalized distance calculation from the pooled residual covariance within group matrix, and clustered with paired group and Manhattan similarity algorithm. Bootstrap support was obtained using 1,000 replicates of the distance matrix. Data were analysed using PAST 1.91 [59] and Statistica [60]. Cavalli-Sforza & Edwards pair-wise genetic distances (Dc) were obtained using the software FreeNA [61]. A neighbour-joining clustered dendrogram based on Dc distances was constructed in Mega 5.1 [62]. One-thousand bootstrap replicates of the Dc matrix were conducted in MSA [63] and used to calculate bootstrap support using PHYLIP 3.69 package [64].

The reproductive investment of each *Cattleya liliputana* population was estimated in the field through establishment of 12, 5 × 2-m plots along a transect placed at the centre of studied populations, and six 5 × 4-m plots randomly distributed throughout the population area, totalling an area of 240 m^2^ per population. The number of pseudobulbs and the number of inflorescence of each individual inside the plots were counted during the 2012 flowering season. The number of flowers per inflorescence was determined on randomly collected pseudobulbs used in the morphometric analysis. The effects of size and genetic clusters identified by STRUCTURE on inflorescence production were tested using a General Linear Model (GLM) on R software [58].

### Correlation Analyses

To test isolation by distance, a Mantel test was performed to determine the correlation between matrices of genetic and geographic distances. In order to verify association between genetic and morphological divergence and between morphological divergence and geographic distances, correlations between distance matrices were estimated. The analyses were performed with 10,000 randomizations in PASSaGE [65]. A Spearman’s rank correlation analysis between the morphological (D2m) and genetic (*H*_e_ and *A*_*R*_) variability was carried out using Statistica 6.1 [60]. D2m was used as a measure of morphological diversity and represents the median of the generalized Mahalanobis distance from individuals to their population centroids.

### Climatic Analyses

To test whether climatic differences may account for population divergence, we compared WorldClim data of major IQ mountain ranges. Random points were created on the study areas above 1,300 m a.s.l. using ArcGis 10.0. The minimum distance between points was set to 500 m, totalling 438 points. Points were separated according to the geographic region: west region (CAL and MOE), south region (OBR), east region (CAP and GAN) and Caraça region (CRP and PSL). Six poorly correlated climatic variables and altitude were extracted from WorldClim data: altitude; annual mean precipitation; annual mean temperature; mean diurnal temperature range; minimum temperature of the coldest month; predicted evapotranspiration; and vegetation index in July, the dry season. Climatic structure of areas was analysed using PCA on Statistica 6.1 [60].

## Results

### Genetic diversity and landscape genetics

All loci were polymorphic with the number of alleles varying from 15 to 39 per locus (S1 Table). MICRO-CHECKER analysis found no evidence of stuttering or allele dropout for any loci, but did suggest the presence of null alleles. Populations showed high diversity with expected heterozygosity, ranging from 0.712 to 0.793, and allelic richness ranged from 6.66 to 11.47. The populations of GAN and CAP had the highest genetic diversity (Table 2). All populations showed deviations from Hardy-Weinberg equilibrium, with *F*_IS_ ranging from 0.250 to 0.350, part of which may be due to the presence of null alleles.

**Table 2:** Parameters of genetic diversity and morphological variability of seven populations of *Cattleya liliputana* based on seven microsatellite loci and 32 continuous floral and vegetative characters.

Populations	*A*_*R*_ (±SD)	*H*_o_ (±SD)	*H*_e_ (±SD)	*D2m*
CAL	9.09 (±3.16)	0.488 (±0.243)	0.753 (±0.210)	19.49
MOE	6.66 (±2.70)	0.537 (±0.227)	0.712 (±0.150)	17.08
OBR	8.23 (±3.25)	0.488 (±0.209)	0.742 (±0.226)	14.73
CAP	9.52 (±3.73)	0.563 (±0.221)	0.781 (±0.189)	19.09
GAN	11.48 (±3.86)	0.534 (±0.175)	0.793 (±0.167)	37.92
CRP	6.93 (±1.75)	0.535 (±0.179)	0.714 (±0.138)	27.02
PSL	7.02 (±3.47)	0.479 (±0.187)	0.714 (±0.169)	21.54

*A*_R_, allelic richness; H_o_, observed heterozigozity; H*e*, expected heterozigozity, D2m, median of the squared Mahalanobis distances of individuals to the population centroid. See Table 1 for populations code.

**Fig 2:**
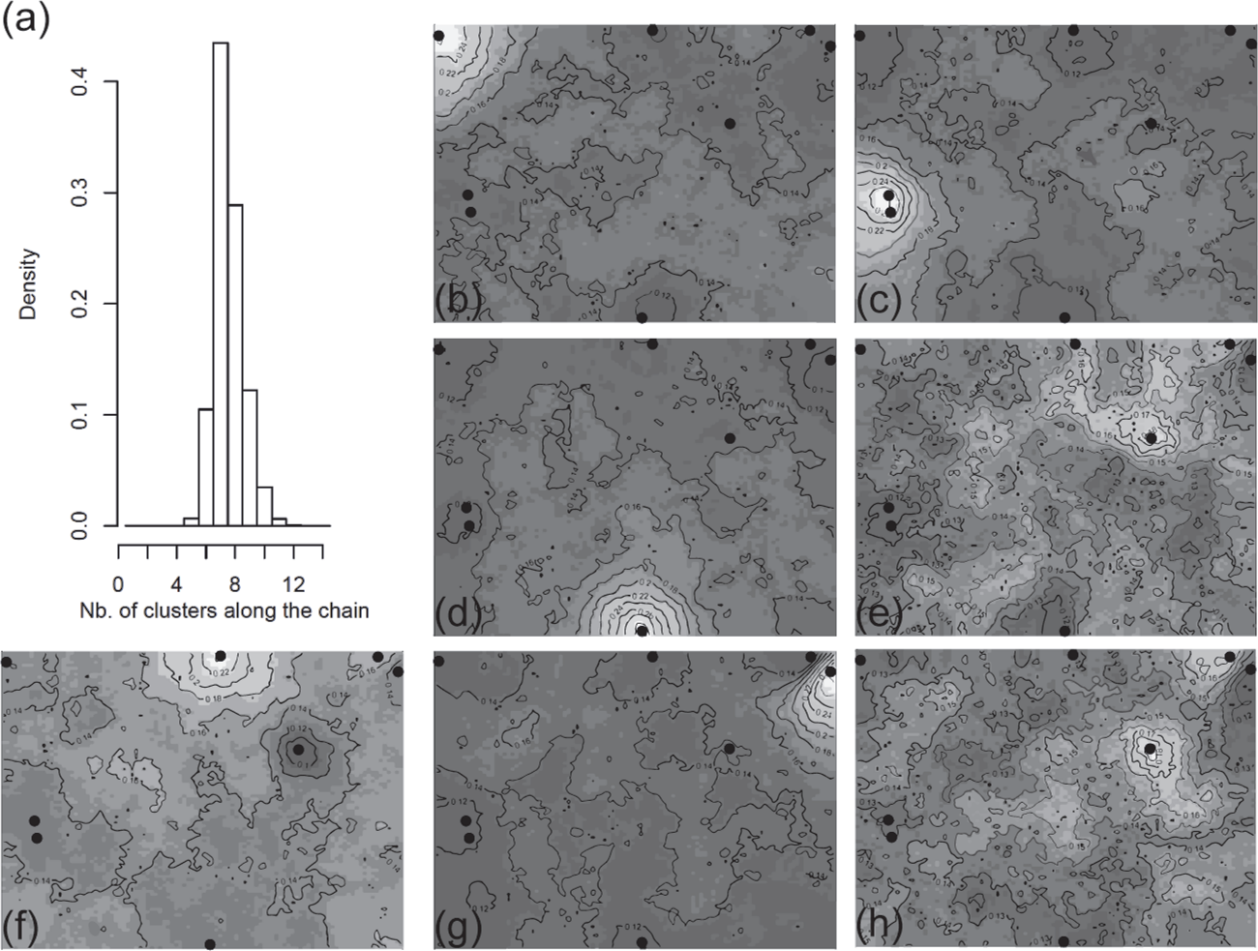
Bayesian clustering analysis conducted in GENELAND. (a) Distribution of posterior probability of a number of *K* genetic clusters. (b–h) Maps of population membership probabilities belonging to one of *K* = 7 clusters for 160 individuals of *Cattleya liliputana*.

Analysis of molecular variance (AMOVA) indicated significant genetic differentiation among populations (*F*_ST_ = 0.138, *p* < 0.001; Table 3). MCMC simulations on Geneland consistently retrieved seven different genetic populations (Fig. 2A). Maps of population membership probability suggest that there is a moderate genetic differentiation among populations even between those in close proximity (Fig. 2B–H). The Mantel test relating genetic and geographical distances between populations was not significant (*r* = 0.371, *p* = 0.071).

**Table 3:** Analysis of Molecular Variance (AMOVA) for different hierarchical levels of seven populations of *Cattleya liliputana*.

Source of variation	Df	Sum of squares	Variance components	% Total variance	*P*-value
*Cattleya liliputana* s.l.					
Among populations	6	9964.55	32.09	13.8	< 0.001
Within populations	313	62524.20	199.76	86.2	< 0.001
Two groups according to Barrier and STRUCTURE: (I) Serra do Caraça group (CRP and PSL) and (II) core IQ (CAL, MOE, OBR, CAP and GAN)					
Among groups	1	5091.65	38.18	15.0	< 0.001
Among populations within groups	5	4763.39	16.07	6.3	< 0.001
Among individuals within populations	313	62633.70	200.11	78.7	0.035
Three groups according to neighbor-joining dendrogram: (I) Serra do Caraça group (CRP and PSL) and (II) east IQ (CAP and GAN) and (III) west IQ (CAL, MOE and OBR)					
Among groups	2	5730.55	18.06	7.6	< 0.001
Among populations within groups	4	4124.50	18.10	7.7	< 0.001
Among individuals within populations	313	62633.70	200.10	84.6	0.07

For groups see Table 1 and Fig. 1.

**Fig 3:**
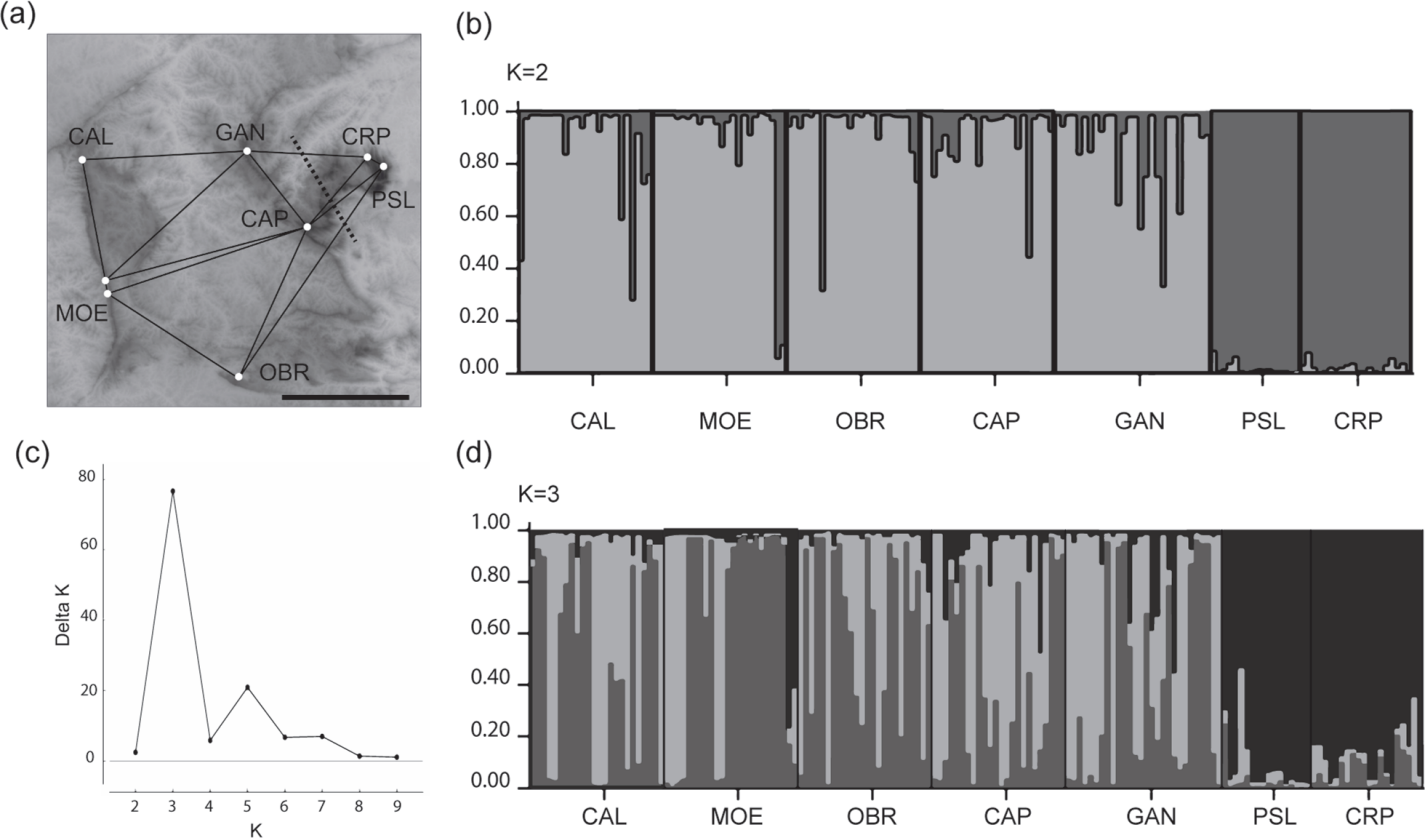
Genetic structure of *Cattleya liliputana* populations. (a) Genetic barrier (dotted line) estimated by Monmonier´s maximum difference algorithm. (b) Representation of Bayesian clustering analysis of seven populations of *Cattleya liliputana* based on seven microsatellite loci for *K* = 2. (c) Delta *K* graphic of the average likelihood for each *K* based on ten runs. (d) Representation of Bayesian clustering analysis of seven populations of *Cattleya liliputana* for *K* = 3. Different colours represent different genetic clusters. Populations are separated by vertical bars. For population names see Table 1.

Monmonier’s analysis revealed one barrier separating Serra do Caraça populations (CRP and PSL) from the remaining populations occurring around the Rio das Velhas valley (core IQ populations) (Fig. 3A). Bayesian simulations carried out on STRUCTURE also suggested a strong differentiation between Serra do Caraça and core IQ populations, consistent with the genetic barrier revealed by Monmonier’s algorithm (Fig. 3B and 3D). ΔK statistics [53] suggested the occurrence of three genetic clusters (Fig. 3C), split into two geographic groups. A second AMOVA clustering the populations into two groups according to STRUCTURE and Monmonier’s algorithms, identified 15% total variation between groups and 6.3% variation among populations within groups (Table 3).

**Fig 4:**
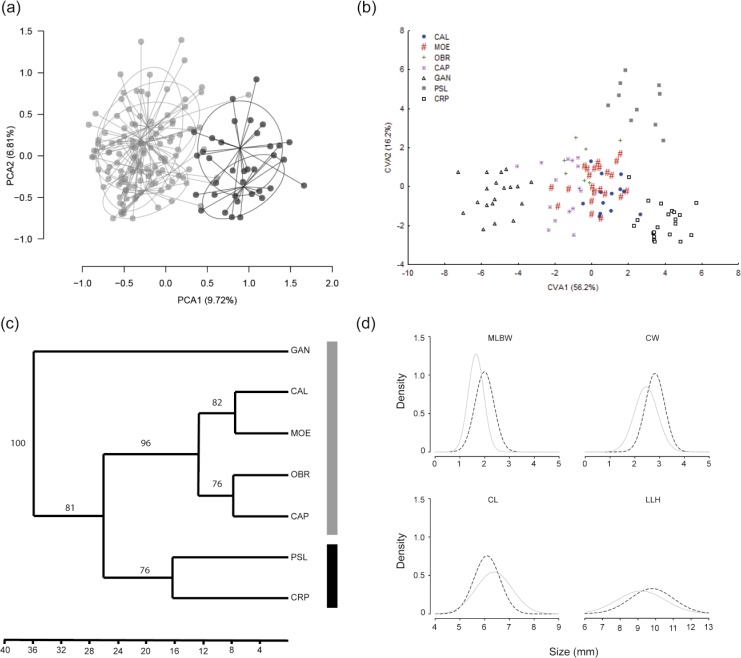
Morphological and genetic divergence of *Cattleya liliputana* populations. (a) Principal component analysis for microsatellite markers of Serra do Caraça (black) and core IQ (grey) individuals. (b) Representation of the scores of first and second canonical axes of CVA using 18 floral and eight vegetative continuous traits. (c) Dendrogram of morphological relationships constructed using Mahalanobis generalized distance clustered with paired group and Manhattan similarity algorithm. Bootstrap support was obtained by 1,000 replicates. Cophenetic correlation = 0.9703. (d) Phenotypic divergence in floral traits for Serra do Caraça (black dashed line) and core IQ (grey solid line). MLBL, medium lobe base width; LLH, lateral lobe high; CW, column width; CL, column length. These characters correspond to numbers 3, 7, 26 and 25, respectively, in S2 Fig. For population names see Table 1.

The PCA performed on population allelic frequencies confirmed that Caraça populations constitute a group strongly differentiated (Fig. 4A). The neighbour-joining dendrogram based on Cavalli-Sforsa pairwise distance (S3 Fig.) also clustered the Caraça populations with strong bootstrap support (99.6%), consistent with previous analyses, and suggested that core IQ populations could be further split into two groups: a western group, including CAL, MOE, and OBR populations, and an eastern group including CAP and GAN populations, with moderate bootstrap support. The AMOVA considering these three genetic groups according to the neighbour-joining dendrogram, showed that 7.6% of total variation was found among groups and 7.7% among populations within groups (*p* < 0.001, Table 3).

### Morphological divergence

The first four axes in the canonical variance analysis (CVA) were statistically significant. The scatterplot of individual scores revealed the separation of the core IQ and Serra do Caraça populations in the first axis, which accumulated 56.2% of total variance (Fig. 4B). There was an overlap of most of the core IQ populations, as seen for the genetic PCA results, with the exception of GAN. The second axis accumulated 16.2% of total variance and showed a morphological structure within the Serra Caraça group.

The paired group dendrogram showing Mahalanobis’ generalized distance of population centroids, revealed a topology of morphological clustering in accordance with genetic findings (Figs. 4C and S3). The correlation between genetic and morphological distances almost reached significance (*r* = 0.422, *p* = 0.053). D2m (morphological diversity) ranged from 14.73 (OBR) to 37.92 (GAN). Population GAN had the highest morphological diversity, consistent with genetic results (Table 2). Genetic diversity was positively correlated with the morphological diversity (Spearman’s correlation for *H*_e_ and D2m, r = 0.306, *p* < 0.05; and for *N*_a_ and D2m, r = 0.285, *p* < 0.05). Morphological distance was not correlated with geographical distance between the populations (Mantel test, *r* = -0.267, *p* = 0.195).

Density plots of column and lip morphometric characters revealed a phenotypic shift between genetic groups. Plants from the Serra do Caraça group produce flowers with wider and taller chambers than do plants from the core IQ group (Fig. 4D). Plants from Serra do Caraça exhibited a larger size for medium lobe base width (MLBL), column width (CW), and lateral lobe height (LLH) than the plants from the core IQ population (all *p* < 0.01). However, column length (CL) was found to be shorter in the Serra do Caraça plants (*p* < 0.01).

### Reproductive investment

**Fig 5:**
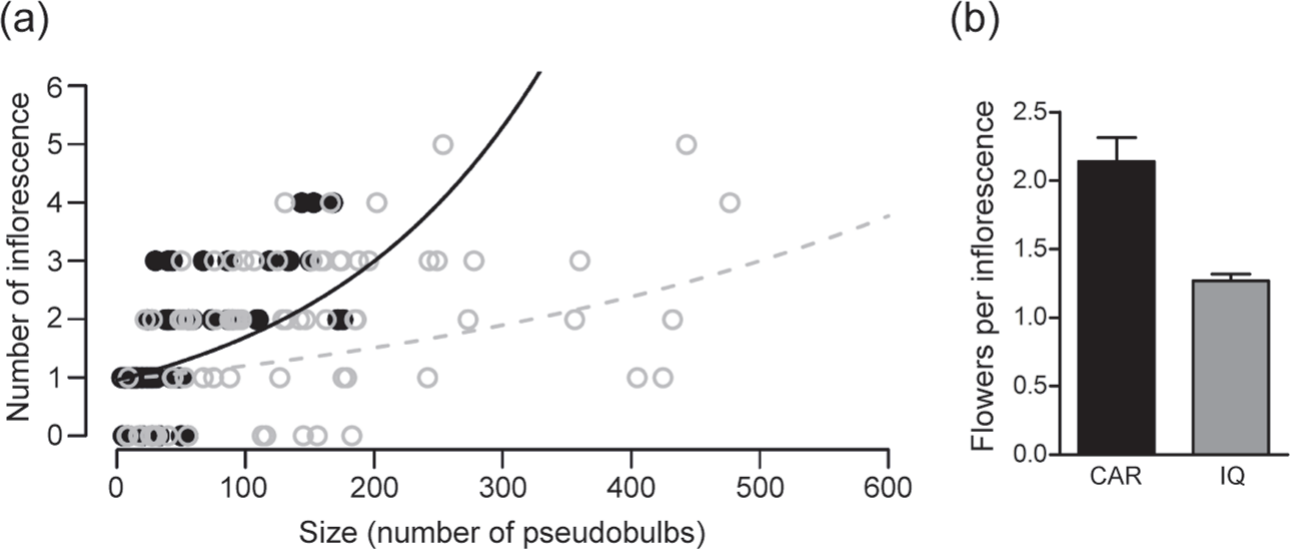
Reproductive investment of *Cattleya liliputana* individuals grouped according to the genetic analysis. (a) Number of inflorescences per size and General Liner Model of inflorescence production for the Serra do Caraça group (black) and the core IQ group (grey). (b) Mean number of flowers per inflorescence. For GLM analysis see S2 Table in the Supporting Information.

General linear model (GLM) analysis indicated a significant positive correlation between individual size (number of pseudobulbs) and production of inflorescences (S2 Table). The mean sizes of plants from the Serra do Caraça and core IQ groups were significantly different (*p* < 0.001, S2 Table). Serra do Caraça individuals bear fewer pseudobulbs than core IQ individuals (size = 56.89 ± 8.97, N = 87 for Caraça, and 160.0 ± 17.05, N = 137 for core IQ individuals). GLM analysis revealed a strong interaction between individual size (number of pseudobulbs) and genetic group, on the number of inflorescences (*p* < 0.001, S2 Table), suggesting that Serra do Caraça populations produce more inflorescences per pseudobulbs than do the core IQ group (Fig. 5A). Furthermore, flower production per inflorescence was also significantly higher for Serra do Caraça (*F* = 5.068, *p* < 0.001) (Fig. 5B). Taken together, these results suggest that there is a trade-off between growth and reproduction, allowing higher investment in inflorescence and flower production for the genetic and phenotypic distinct populations at Serra do Caraça.

### Climatic niche analysis

**Fig 6:**
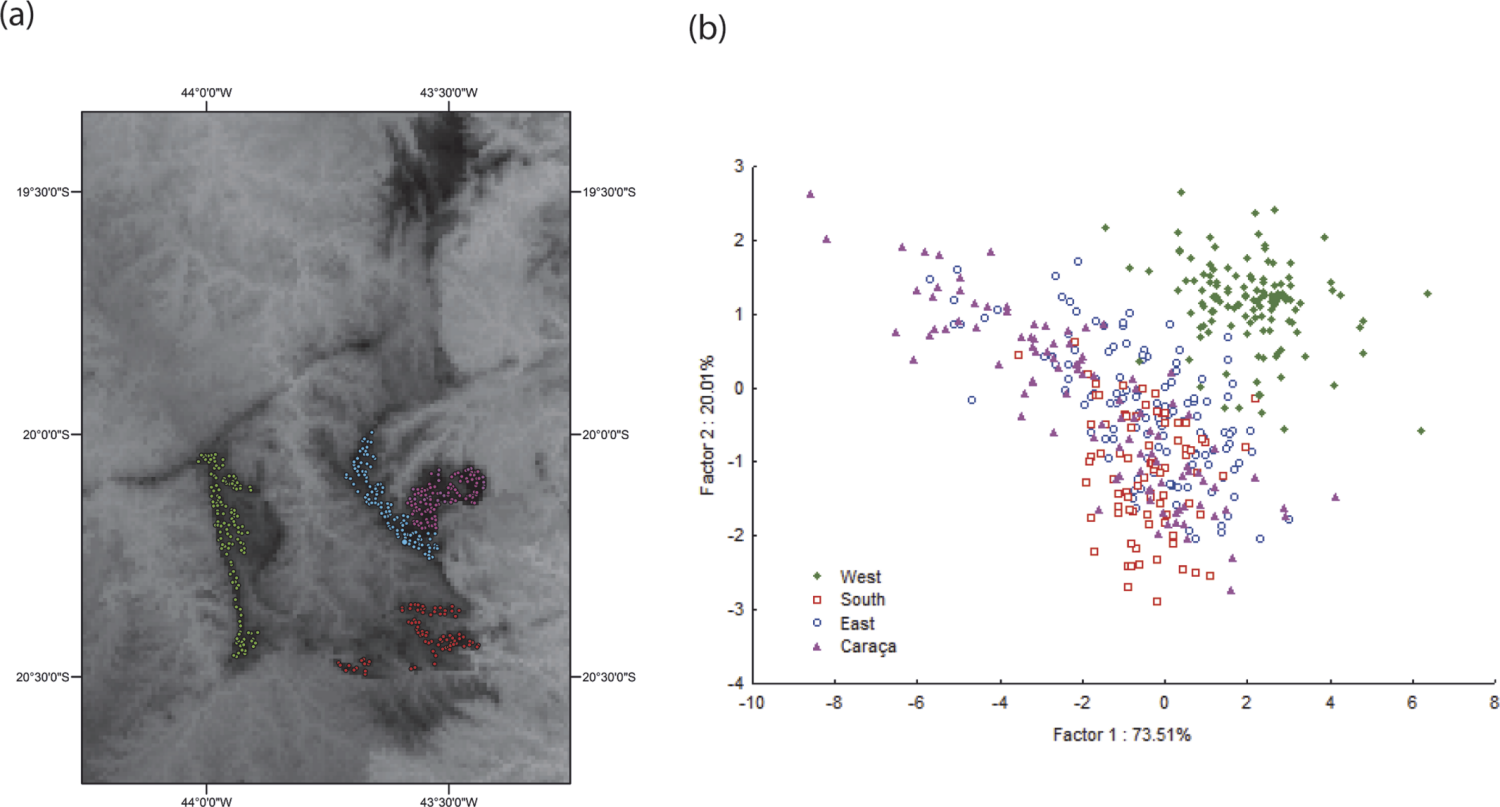
Climatic structure of mountain areas above 1,300 m a.s.l. in the Iron Quadrangle, Brazil. (a) Random points sampled for climatic variables are coloured according to geographic region; Green, west region; Red, south region; Blue, east region; Pink, Serra do Caraça region. (b) Principal component analysis of sampled points according to annual mean precipitation; annual mean temperature; mean diurnal temperature range; minimum temperature of the coldest month; predicted evapotranspiration; vegetation index at July and altitude.

In order to test whether climatic differences may account for the differences in genetic, morphological, and reproductive investment between the Caraça and core IQ groups, we compared WorldClim data of the major IQ mountain ranges (Fig. 6). PCA of randomly seeded points at areas above 1,300 m a.s.l., the *Cattleya liliputana*-populated area, revealed that there is climatic heterogeneity between IQ regions (Fig. 6A). However, the climatic pattern is not consistent with the observed genetic differentiation. Thus, current macroclimatic differences do not support a climatic hypothesis for population divergence, suggesting that other factors, which were unaccounted for in this analysis (e.g. microclimatic differences and rock type), may play important roles in population divergence in this region.

## Discussion

### Genetic diversity and structure based on microsatellite loci

Genetic studies of montane species can provide insights into the combined effects of genetic drift, restricted gene flow, and local adaptation on the evolution of plant lineages restricted to disjoint populations [13,14,18]. In *C*. *liliputana*, the absence of a pattern of isolation by distance is suggestive of the presence of geographical or ecological barriers to gene flow. Bayesian modelling of population membership probability and AMOVA results showed a significant genetic differentiation in accordance with outcrop and mountain discontinuities suggesting that landscape contributes to genetic isolation at outcrop environments, even at short distances. Genetic studies of rock outcrop species have indeed confirmed some hypotheses associated with such habitats, such as low levels of gene flow and strong genetic drift [14,21,66–68].

The most significant pattern revealed by the use of microsatellite markers was the deep population differentiation between the Serra do Caraça and core IQ groups. A combination of genetic analyses revealed a similar population structure. Bayesian modelling of microsatellite data using STRUCTURE, Monmonier´s maximum difference algorithm, PCA of population allelic frequencies, and neighbour-joining tree based on pairwise genetic differentiation, identified the presence of one major barrier separating populations of quartzite outcrops of Serra do Caraça from the core IQ populations predominantly at iron outcrops. A less remarkable barrier separating the west and east populations of the core IQ was detected by a neighbour-joining tree, suggesting that the Rio das Velhas valley can constitute a partial barrier to gene flow. AMOVA corroborated these analyses, showing a high differentiation between the Serra do Caraça and the core IQ populations (F_CT_ = 0.15), and moderate differentiation (F_CT_ = 0.076) between the three groups, Serra do Caraça, the west and east IQ. Populations CAP and GAN, in the transition zone between Serra do Caraça and core IQ genetic clusters, showed the highest diversity and admixture, suggesting that gene flow may occur through these locations in a stepwise manner before the western mountains are reached.

Nuclear genetic differentiation of orchids tends to be low when compared with other plant taxa [69]. The genetic structure at a landscape scale showed by *C*. *liliputana* can be considered high (F_ST_ = 0.138) in comparison with other orchid species on Neotropical mountains. Studies over a larger spatial scale using *Cattleya* species resulted in similar [47,70] and lower [71] fixation indices. The genetic structure of *C*. *liliputana* is also considerably higher than that found for *Laelia* and *Epidendrum* [14,72,73] and higher than the overall *F*_ST_ values found for the myophilous *Bulbophyllum* and *Acianthera* species on the northern Espinhaço mountains [74,75].

Limited gene flow across the top of mountain showed by *C*. *liliputana* may be due to low pollinator movement. *C*. *liliputana* is one of the smallest species in the *Cattleya* genus, restricting the pollinator size to get access to the flower chamber formed by the bending of the lip towards the column. *C*. *liliputana* flowers are visited by small solitary bees [76]. Small bees are known to have low movement dispersion [77]. Moreover, the high endemism exhibited by most of *Cattleya* series *Parviflorae* species [42] suggests strong constrains on seed germination and recruitment, possibly associated with local microhabitat demands, what may limit the capability of species dispersion, thus reinforcing the effects of isolation.

### Morphological divergence and reproductive trade-offs: evidence of local adaptation?

Morphometric analysis of *C*. *liliputana* flower and vegetative traits revealed a similar grouping pattern to that found using genetic markers, with a remarkable separation of Serra do Caraça plants from the remaining populations (Fig. 4A–C). PSL and CRP populations from Serra do Caraça showed phenotypic shifts in morphometric traits that define the size of the flower chamber. The wider and taller pollinator chambers (Fig. 4D) result in a larger entrance for pollinators. This phenotypic shift may allow these populations to exploit new pollinators. Indeed, changes in the column length are known to change the site of pollinaria deposition and avoid hybridization between sympatric *Cattleya* species [78]. Similar changes found for *C*. *liliputana* could reduce gene flow and enhance genetic divergence between Serra do Caraça and IQ populations.

Pollinator-mediated divergent selection is thought to promote speciation in several plant groups [79]. However, generalized food-deceptive pollination systems usually attracts a wide range of occasional pollinators that, after a few trial visits, avoid the flowers and switch to more rewarding ones [80]. This insect behaviour exerts only a weak selective pressure on floral traits [81–83] that is unlikely to produce effective premating incompatibilities. However, this may not be the case for montane food-deceptive *Cattleya* species, which are pollinated by few pollinators. In this pollinator system, morphological changes in flower traits were found to be an important premating barrier for reproductive isolation of sympatric species [78,84].

Serra do Caraça populations also exhibited higher individual investment in the production of inflorescences and the number of flowers per inflorescence. The increase of sexual reproductive effort may be a response to the lower mean individual size (Fig. 5), which suggests adverse conditions for growth and vegetative reproduction. Investment in reproduction is associated with the ability of the population to survive under strong selective pressures. For example, populations colonizing new environments are selected on the basis of increased reproductive effort [85], because even small increases in reproductive rate are able, over time, to result in substantially larger population growth.

The variation in reproductive strategy and flower traits between populations at different rock types suggest that local adaptation could be involved in the observed differentiation between Serra do Caraça and the remaining populations. Metal-rich soils are known to promote local adaptation as observed for *Arabidopsis*, *Helianthus* and *Pinus* [86–88]. Moreover, flower size and morphology were shown to be highly heritable characters in many plant species, and can thus respond to natural selection [89–92]. The genetic basis of flowering and the balance of growth and reproductive investment have also been traced to genes and quantitative trait loci (QTL) in model plants [93–95]. Furthermore, phenotypic plasticity can also explain part of the variation in reproductive strategy and morphology found in *C*. *liliputana*. Regulatory gene networks responsive to environmental cues and plasticity in the expression of adaptive genes may be important sources of adaptability to environmental heterogeneity [96]. The genetic accommodation, the evolution of increased or decreased levels of plasticity, can act to fine tune the adaptability at the microenvironment scale. However, no information on the genetic basis of flower morphology or reproductive investment is available for our study species. Although our results are consistent with an adaptive scenario, we acknowledge that the phenotypic divergence we observed could also have a contribution of non-adaptive genetic variation, i.e, genetic drift. Nonetheless, transcriptome and gene expression studies in association with reciprocal transplants are under way to test the relative role of phenotypic plasticity and local adaptation in *C*. *liliputana* population divergence [97,98] and improve the understanding of evolution in rock outcrop landscapes.

### Are populations of *C*. *liliputana* undergoing speciation?

Population differentiation has long been perceived as an early step during the speciation process [99]. The genetic, morphological, and reproductive differences found here open the debate about the degree of divergence between these groups, raising the question whether *C*. *liliputana* populations are undergoing speciation.

From a broader perspective, the relationship between population divergence and speciation is not always obvious. Indeed, some species can maintain high population differentiation without necessarily splitting into several lineages [100]. Conversely, speciation can sometimes emerge in the presence of gene flow [101,102], in particular, when the diverging populations become highly adapted to their respective habitats [103–105]. *C*. *liliputana* populations from Serra do Caraça have been referred to as *Cattleya aff*. *kettiana* by some plant taxonomists [106]. However, although Serra do Caraça populations are genetically and morphological differentiated, they share most of alleles with core IQ populations, what do not support the idea that the two genetic groups are reproductively isolated. Further research on reproductive biology and reciprocal crossing experiments will aid our understanding of the mechanisms driving speciation in this orchid lineage.

### Environmental landscape

We investigated some putative environmental factors that may elicit divergent selective pressures in the IQ landscape (Fig. 6). IQ highlands make up most of the climatic gradient that separates the Cerrado biome in the west, and the Atlantic Rain Forest biome in the east. The climatic analysis of high-altitude areas in the IQ revealed that mountain ranges are heterogeneous and substantially different from each other. However, the genetic and ecological divergence of the Serra do Caraça populations cannot be fully explained by landscape climatic differences. Microclimatic differences and rock outcrop type are probably the major selective pressures. For instance, these regions vary greatly in outcrop composition. Iron outcrops are mostly found around the Rio das Velhas valley being almost absent in the Serra do Caraça region. Quartzite outcrops at Serra do Caraça are large massive rocks that extend over several kilometres while most of the iron outcrops around the Rio das Velhas valley are small and intercalated with patches of forest and grassland. In accordance with *C*. *liliputana*, genetic differentiation of *Vellozia compacta* was also found to be associated with the disjunct distribution of populations at different rocky types (iron vs. quartzite) [107], suggesting that common environmental factors such as rock type, could act broadly in the evolutionary process in outcrop vegetation communities.

### Implications for conservation

One environment particularly threatened by climate change is the Neotropical mountain area [10,11,108]. However, there are very little data available to support efficient managing initiatives in these environments. The genetic and ecological divergence of *C*. *liliputana* found here is likely to influence the long-term success of managing efforts, as the genetic differentiation and local adaptation might aid or hinder the survival and reproduction of managed plants. Taking into account the genetic, morphological, and reproductive divergence of *C*. *liliputana*, we recommend that Serra do Caraça and core IQ populations should be managed separately as different evolutionary significant units. Conservation of *C*. *liliputana* would also benefit from the rescue of populations in areas directly affected by mining and oriented reintroduction in accordance with genetic background in order to minimize population impact of habitat degradation in the IQ.

## Supporting Information

S1 Fig Representative habitat and individuals of *Cattleya liliputana*. (a) Iron outcrop and (b) quartzite outcrop at high-altitude areas of the Iron Quadrangle. (c) Representative *C*. *liliputana* individual growing on an iron outcrop. (d) Representative *C*. *liliputana* individual growing on a quartzite outcrop.
(TIF)

S2 Fig Outline of flower and vegetative parts indicating the morphological characters used in the morphometric analysis. (TIF)

S3 Fig Dendrogram of Cavalli-Sforsa pairwise genetic distance with supporting values based on 1,000 bootstrap replicates. (TIF)

S1 Table Microssatelite primers, amplification conditions and allele richness of 160 individuals of *Cattleya liliputana*. (DOCX)

S2 Table General Linear Model for size and number of inflorescence. (DOCX)
