# Supplementary figures and images for: Genetic Structure Is Associated with Phenotypic Divergence in Floral Traits and Reproductive Investment in a High-Altitude Orchid from the Iron Quadrangle, Southeastern Brazil
Source: PLoS One. 2015 Mar 10;10(3):e0120645. doi: 10.1371/journal.pone.0120645 (PMC4355488; doi:10.1371/journal.pone.0120645)

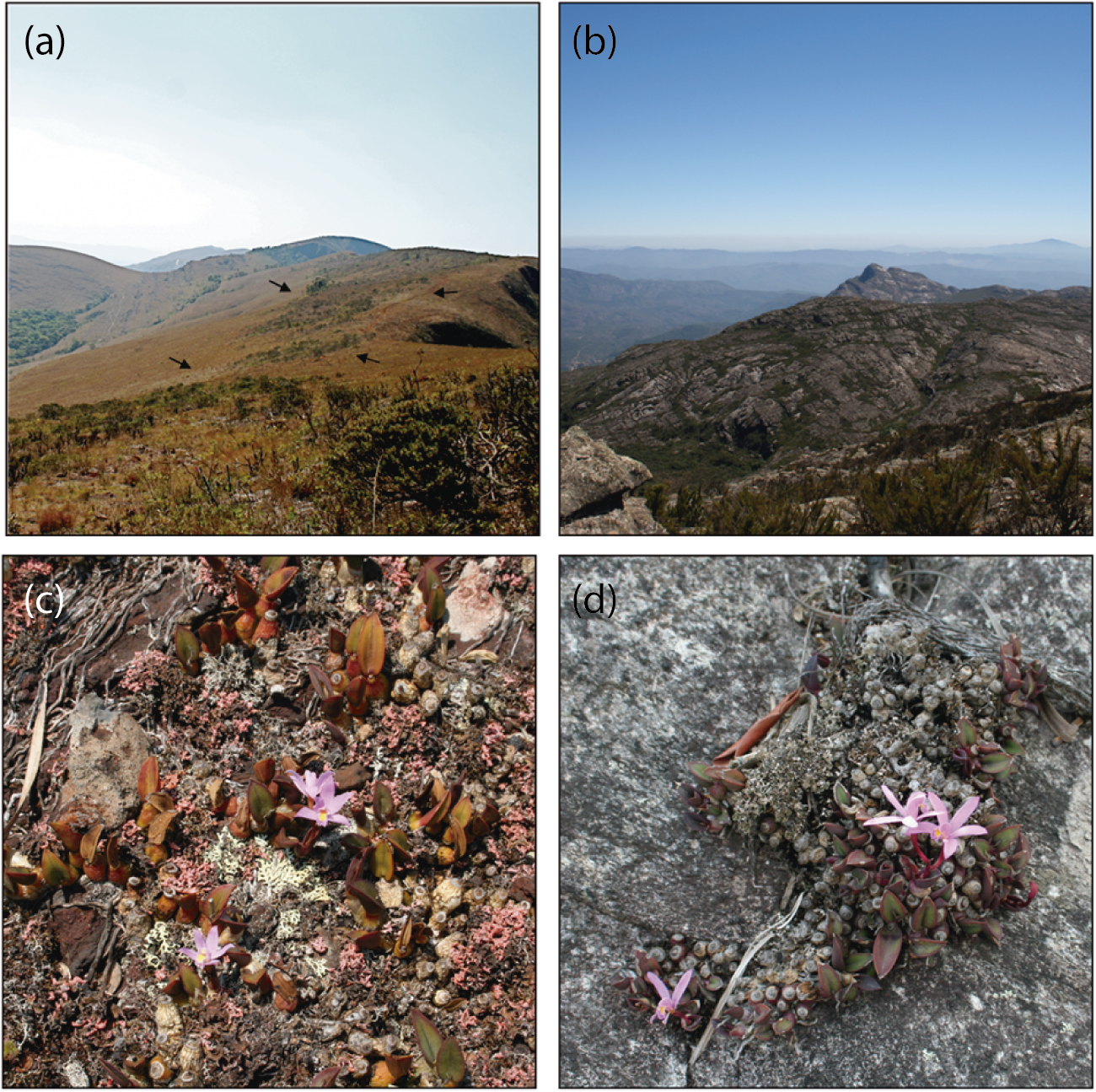

Supplement: S1 Fig — (a) Iron outcrop and (b) quartzite outcrop at high-altitude areas of the Iron Quadrangle. (c) Representative C. liliputana individual growing on an iron outcrop. (d) Representative C. liliputana individual growing on a quartzite outcrop. (TIF) [file pone.0120645.s001.tif]

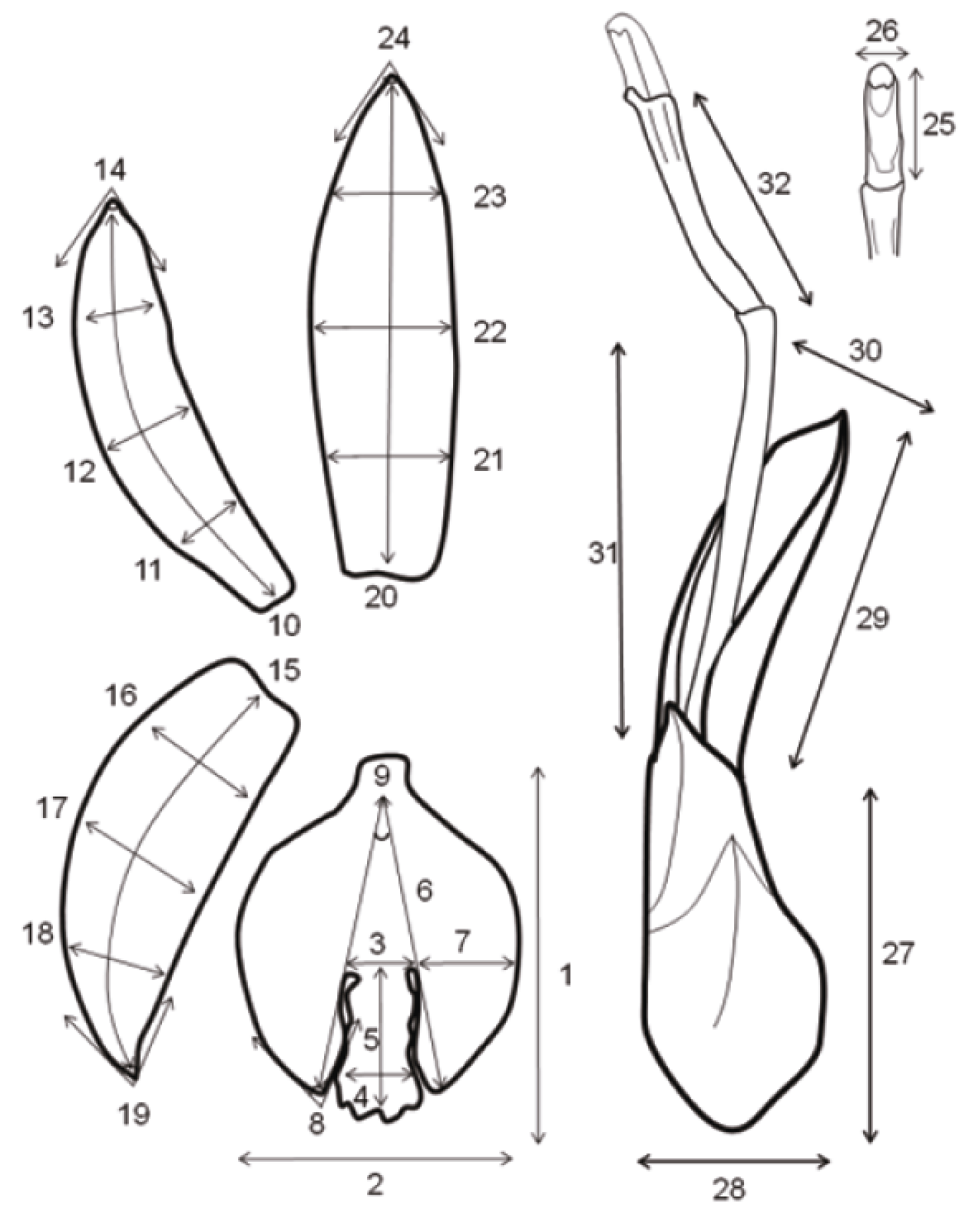

Supplement: S2 Fig — (TIF) [file pone.0120645.s002.tif]

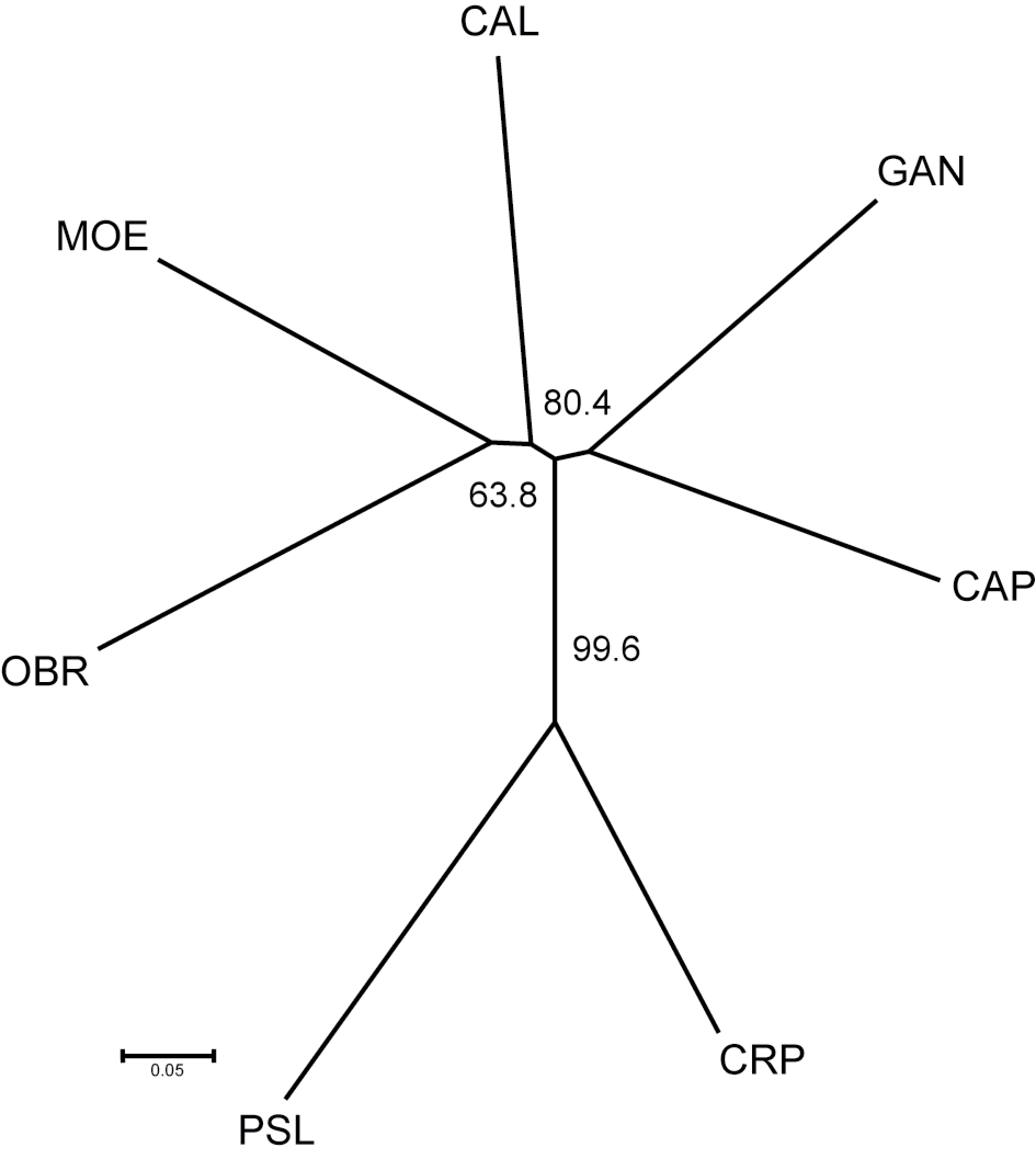

Supplement: S3 Fig — (TIF) [file pone.0120645.s003.tif]
