# Supplementary material for: Genetic Structure Is Associated with Phenotypic Divergence in Floral Traits and Reproductive Investment in a High-Altitude Orchid from the Iron Quadrangle, Southeastern Brazil
Source: PLoS One. 2015 Mar 10;10(3):e0120645. doi: 10.1371/journal.pone.0120645 (PMC4355488; doi:10.1371/journal.pone.0120645)
Supplement: S1 Table — (DOCX) [file pone.0120645.s004.docx]

| **S1 Table . Microssatelite primers, amplification conditions and allele richness of 160 individuals of *Cattleya liliputana***. | | | | | | |
| --- | --- | --- | --- | --- | --- | --- |
| Locus | Primer sequence (5'-3') | Repeat motif | *T*_a_ (°C) | Size (bp) | *N*_a_ | Source |
| Cac01 | F: M13-TACAACGCCCAATTTGAATG | (GA)17 | 60.0 | 108 | 31 | Novello *et al*., 2013 |
|  | R: CCATCATTTGCCTTTTCACA |  |  |  |  |  |
| Cac02 | F: M13-CAGGATTTCTCCTCGTGCTC | (AG)18 | 60.0 | 173 | 29 | Novello *et al*., 2013 |
|  | R:GCAGAGCGGAACAAGGATAG |  |  |  |  |  |
| Cac11 | F: M13-TCAAGGCCTGCACATAGAGA | (AG)8 | 60.0 | 167 | 15 | Novello *et al*., 2013 |
|  | R: AAGAGGAAGGCTTCGTTGC |  |  |  |  |  |
| Cac16 | F: M13-AACAGGCATTTGGAGCTTTT | (AG)23 | 60.0 | 250 | 39 | Novello *et al*., 2013 |
|  | R: CCTCATTTCTCTCACCCTCTTT |  |  |  |  |  |
| Cac18 | F: M13-CTGGTGAGGGAGAAGAAAAACA | (GA)11N(AG)26 | 60.0 | 224 | 28 | Novello *et al*., 2013 |
|  | R: CCCTCTCCCTCTCTTTTCCA |  |  |  |  |  |
| Cac26 | F: M13-TGGCTTGGTGTTGCATTTAT | (GA)6(GT)6 | 56.0 | 167 | 28 | Leal, 2013 |
|  | R: TGTGAGCCTCAATAAGCCAAT |  |  |  |  |  |
| Cac27 | F: M13-TGGATCTTACCTTGGGCTTCTA | (GT)7GC(GT)4(GA)11 | 62.0 | 209 | 30 | Leal, 2013 |
|  | R: CCCTGCTCTCTTTCTCACTCAT |  |  |  |  |  |
| M13-Tail: TTTTCCCAGTCACGAC;  *T*_a_ temperature of annealing; *N*_a_ allele richness | |  |  |  |  |  |
