# Supplementary material for: Genetic Structure Is Associated with Phenotypic Divergence in Floral Traits and Reproductive Investment in a High-Altitude Orchid from the Iron Quadrangle, Southeastern Brazil
Source: PLoS One. 2015 Mar 10;10(3):e0120645. doi: 10.1371/journal.pone.0120645 (PMC4355488; doi:10.1371/journal.pone.0120645)
Supplement: S2 Table — (DOCX) [file pone.0120645.s005.docx]

| **S2 Table. General Linear Model for size and number of inflorescence.** | | | | | | | |
| --- | --- | --- | --- | --- | --- | --- | --- |
|  | Variable | Df. | Deviance | Residual Df. | Residual Dev. | F | p |
| Size |  |  |  |  |  |  |  |
|  | Genetic group | 1 | 3971.2 | 156 | 9376.6 | 58.52 | p<0.001 |
| Number of inflorescence |  |  |  |  |  |  |  |
|  | Size | 1 | 27.452 | 156 | 129.31 | 47.69 | p<0.001 |
|  | Genetic group | 1 | 0.253 | 155 | 129.06 | 0.44 | p = 0.5078 |
|  | Size : Genetic group | 1 | 10.472 | 154 | 118.56 | 18.19 | p<0.001 |
